# Improving the precision of depression diagnosis in general practice: a cluster-randomized trial

**DOI:** 10.1186/s12875-021-01432-w

**Published:** 2021-05-07

**Authors:** Ursula Ødum Brinck-Claussen, Nadja Kehler Curth, Kaj Sparle Christensen, Annette Sofie Davidsen, John Hagel Mikkelsen, Marianne Engelbrecht Lau, Merete Lundsteen, Claudio Csillag, Carsten Hjorthøj, Merete Nordentoft, Lene Falgaard Eplov

**Affiliations:** 1 Copenhagen Research Center for Mental Health, Mental Health Center Copenhagen, Gentofte Hospitalsvej 15, 2900 Hellerup, Denmark; 2 Research Unit for General Practice, Institute of Public Health, Aarhus University, Bartholins Allé 2, 8000 Aarhus C, Denmark; 3 The Research Unit for General Practice and Section of General Practice, University of Copenhagen, Oester Farimagsgade 5, Postbox 2099, 1014 Copenhagen K, Denmark; 4 Mental Health Center Frederiksberg, Nordre Fasanvej 57-59, 2000 Frederiksberg, Denmark; 5 Stolpegaard Psychotherapy Center, Stolpegaardsvej 20, 2820 Gentofte, Denmark; 6 General Practitioner in Copenhagen, Copenhagen, Denmark; 7 Mental Health Center North Zealand, Dyrehavevej 48, 3400 Hilleroed, Denmark

**Keywords:** Depression, Identification of depression, Primary Health Care, General practice

## Abstract

**Background:**

Methods to enhance the accuracy of the depression diagnosis continues to be of relevance to clinicians. The primary aim of this study was to compare the diagnostic precision of two different diagnostic strategies using the Mini International Neuropsychiatric Interview (MINI) as a reference standard. A secondary aim was to evaluate accordance between depression severity found via MINI and mean Major Depression Inventory (MDI) sum-scores presented at referral.

**Methods:**

This study was a two-armed, cluster-randomized superiority trial embedded in the Collabri trials investigating collaborative care in Danish general practices. GPs performing case-finding were instructed always to use MDI when suspecting depression. GPs performing usual clinical assessment were instructed to detect depression as they would normally do. According to guidelines, GPs would use MDI if they had a clinical suspicion, and patients responded positively to two or three core symptoms of depression. We compared the positive predictive value (PPV) in the two groups.

**Results:**

Fifty-one GP clusters were randomized. In total, 244 participants were recruited in the case-finding group from a total of 19 GP clusters, and 256 participants were recruited in the usual clinical assessment group from a total of 19 GP clusters. The PPV of the GP diagnosis, when based on case-finding, was 0.83 (95% CI 0.78–0.88) and 0.93 (95% CI 0.89–0.96) when based on usual clinical assessment. The mean MDI sum-scores for each depression severity group indicated higher scores than suggested cut-offs.

**Conclusions:**

In this trial, systematic use of MDI on clinical suspicion of depression did not improve the diagnostic precision compared with the usual clinical assessment of depression.

**Trial registration:**

The trial was retrospectively registered on 07/02/2016 at ClinicalTrials.gov. No. NCT02678845.

**Supplementary Information:**

The online version contains supplementary material available at 10.1186/s12875-021-01432-w.

## Background

Depression is a common mental disorder in Denmark [1], and according to the World Health Organization, the largest contributor to disability worldwide [2]. For patients with depression, the first point of contact with the health care system is usually in primary care, and most patients with depression are treated in this setting without being referred. However, patients with mental disorders often present with somatic symptoms [3] and social problems, and their depressive symptoms can fluctuate and be mixed with, for example, anxiety symptoms, which can make the diagnostic process difficult [4]. Research indicates that general practitioners (GPs) identify about 47% of depressed patients [5], and a meta-analysis has found, based on data from a subsample of 19 studies, that for every 100 unselected cases assessed for depression in primary care, there were more false positives than either identified or missed cases [5]. Early research failed to show that notification of depression status and education of GPs in identifying depression had an effect on patient outcomes [6, 7]. Other studies have found early identification of depression as a predictive factor of better treatment outcomes [8]. On this background, enhancing the detection of depression in primary care continues to be of importance. Guidelines in the US recommend routine screening for depression [9] in contrast to guidelines in the UK and Canada [10]. In Denmark, a depression screening tool is recommended for use in high-risk groups [11]. However, this is not supported by the literature [12–14]. A randomized controlled trial (RCT) evaluating the effectiveness of screening for major depressive disorder in high-risk groups in primary care found no difference in recognition rates in the screening group compared to the control group [12]. In a prospective cohort study investigating screening in high-risk groups, only 1% started treatment for major depressive disorder as a result of screening [13].

Systematic identification of patients with depression is an active ingredient in collaborative care [15], which is an effective way of managing depression in primary care [16]. Mandatory use of a diagnostic tool when suspecting depression could, therefore, be an appropriate way of improving accurate diagnostics of depression in general practice. A Danish study investigating high-risk screening for depression compared with case-finding (use of a validation instrument on clinical suspicion of depression) found that screening in high-risk groups had limited effect in addition to case-finding, where the GP used Major Depression Inventory (MDI) [14]. However, this observational study had some limitations because the GPs were free to perform either high-risk screening or case-finding, which was not compared with usual clinical assessment. Based on the above literature, case-finding, where the GP *always* uses a validation test on clinical suspicion of depression, may be as good as high-risk screening, but it is unclear if case-finding is better than usual clinical assessment, where a validation tool is used when the GP finds it appropriate. Therefore, a well-planned RCT is needed to examine if case-finding is more effective in finding depression than usual clinical assessment.

## Methods

### Objectives

The primary aim was to determine if case-finding with the mandatory use of MDI on clinical suspicion was more accurate than usual clinical assessment in identifying depression in Danish general practice. The hypothesis was that case-finding would be more accurate than usual clinical assessment. Accuracy was estimated based on the positive predictive value (PPV) in the two groups using the MINI International Neuropsychiatric Interview (MINI) [17] as a reference standard. A secondary aim was to evaluate the accordance between depression severity found via MINI and mean MDI sum-scores.

### Design

**Fig. 1:**
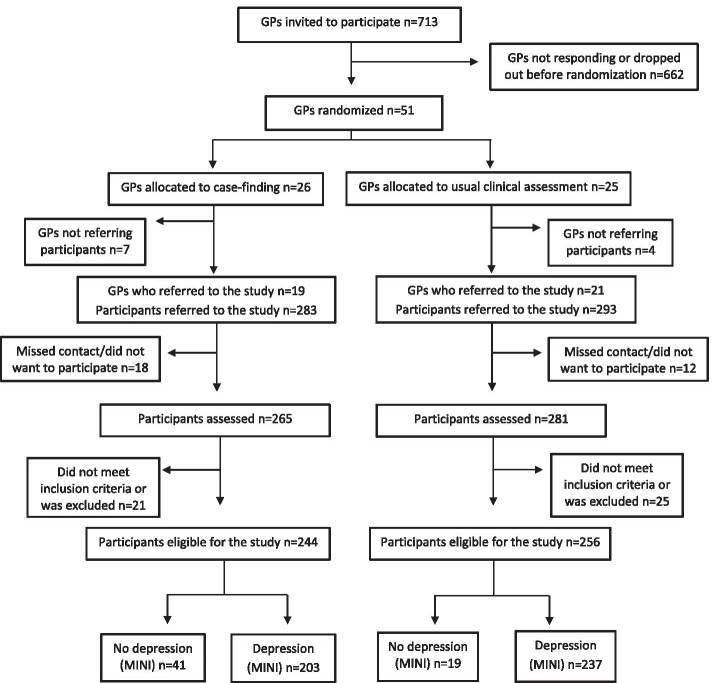
Flow chart

The study was set up as a two-armed, cluster-randomized clinical superiority trial with an intervention group (case-finding) and a control group (usual clinical assessment). It was nested within the Danish Collabri trials investigating collaborative care for depression and anxiety disorders in primary care [18–20] (see Fig. 1). The Collabri trials refer to the Collabri studies and Collabri Flex studies. These studies investigated a complex intervention with a multi-professional approach to management and treatment of depression and anxiety. The intervention consisted of multiple components such as supervision by a psychiatrist and the introduction of a care manager to collaborate with GPs in providing evidence-based care. The control group in the Collabri studies received treatment as usual (TAU), whereas the GPs providing care for the control group participants in the Collabri Flex studies could also consult a team of mental health specialists.

### Participants

GPs in the Capital Region of Denmark (except the Island of Bornholm) were invited to participate in the study. GPs were recruited from May 2014 to July 2015, and patients were recruited from November 2014 to July 2018. Cluster-randomization was conducted using a centralized random computer-generated allocation sequence, carried out externally by the Research Centre for Prevention and Health. Each cluster corresponded to a GP provider number. A provider number could include one or more GPs. First, cluster-randomization for collaborative care intervention was performed. This randomization was performed in three rounds. The allocation ratio was 1:1 in the first two rounds and 3:1 (control:intervention) in the third, including four GPs. Clusters in each group were randomized (allocation ratio 1:1) according to the depression detection method, either case-finding or usual clinical assessment. Many GPs from the Collabri studies also participated in the following Collabri Flex studies, where they kept their detection allocation. We planned only to include participants from the Collabri studies; however, due to limited participant intake, the recruitment strategy was changed to also include participants from the Collabri Flex studies.

The GPs who agreed to participate identified potential participants with depression in their practice and referred them to the Collabri trials. This nested study used data from the referral process and the eligibility interviews. Patients were included if the GP had diagnosed depression, if they were 18 years or older, Danish-speaking, had given their written consent and did not meet any exclusion criteria. Only some of the exclusion criteria from the Collabri and Collabri Flex studies [18, 20] pertained to this nested study. Exclusion criteria included pregnancy, a dementia diagnosis, and having an unstable somatic condition as determined by the GP. Additionally, patients of GPs allocated to collaborative care could not participate if they preferred treatment through the publicly subsidized psychologist program. For participants referred from the Collabri studies current/past (within six months) medical/psychological treatment for anxiety or depression and having a pending disability pension application were also exclusion criteria. The in-and exclusion criteria are updated from earlier descriptions [18].

### Identification of depression

In the usual clinical assessment group, GPs were instructed to diagnose depression as they would normally do [18]. In accordance with National guidelines, GPs are recommended to explore a patient’s condition further using the MDI or ICD-10 criteria if at least two core symptoms of depression, according to ICD-10, are present [21]. In the case-finding group, GPs were instructed to systematically apply the MDI every time they suspected depression and to let the MDI result guide the diagnostics process. Usually, within a week from GP’s referral, a research assistant conducted the MINI interview for DSM IV diagnoses [17] and asked ICD-10 specific questions to validate the ICD-10 diagnosis of depression. Depression severity was assessed using ICD-10. Assessors were blinded for GP allocation and, in the Collabri studies, for the participant’s referral diagnosis. As the study was nested within the Collabri trials, GPs could also refer patients with anxiety disorders who were not eligible for this nested study. Assessors were trained in the MINI interview and received ongoing support and supervision within the research team. If the MINI diagnosis differed from the GP diagnosis, a psychiatrist within the project group would be consulted, and the GP was contacted to determine the result. In case of discrepancy, the GP took the final decision.

### The Major Depression Inventory (MDI)

The MDI consists of 12 items, each item scored on a six-point Likert scale. Used as a diagnostic tool, two out of three of the top three items corresponding to ICD-I0 depression diagnosis’s core symptoms must be present most of the time or all the time for two weeks. At least two of the remaining seven items (two items have subitems where only the item with the highest score counts) corresponding to the accompanying symptoms of the ICD-I0 depression diagnosis must be present more than half of the time the same period to suggest depression. Hereafter the ICD-10 algorithms establish whether the depression is mild, moderate, or severe [21]. The MDI has been validated in an outpatient sample of 43 participants showing an acceptable sensitivity ranging from 0.86 to 0.92 and a specificity ranging from 0.82 to 0.86 [22], and the tool has been used to determine the presence of depression in clinical settings [23, 24].

### The MINI International Neuropsychiatric Interview (MINI)

The MINI is a short and structured diagnostic interview [17]. It has been validated in relation to the Structured Clinical Interview for DSM-III-Revised Patients (SCID-P) with a kappa-value for major depression of 0.83, a sensitivity of 0.96, a specificity of 0.88, a PPV of 0.87, and a negative predictive value (NPV) of 0.97 [17]; and in regards to the Composite International Diagnostic Interview (CIDI) for International Statistical Classification of Disease (lCD) with a kappa-value for major depression of 0.73, a sensitivity of 0.94, a specificity of 0.79, a PPV of 0.82, and an NPV of 0.93 [25].

The MINI was chosen as a reference standard in the present study. Initially, we investigated whether it could be managed over the telephone. Details and results of this validation are shown in Box 1 in Additional file 1.

### Statistical methods

Initial sample size calculations showed that a minimum of 480 participants should be included [18]. Based on the study by Mitchell et al., the PPV for usual clinical assessment can be set at 45% [5]. A clinical possible and meaningful increase is assessed to be 60%. Thus, we wanted to detect a difference in PPV of 15%, with an alpha of 0.05, a power of 0.8, a cluster-size of 10, and an ICC of 0.04.

In the primary analyses, the PPV of GP depression diagnosis in the two detection groups was calculated by constructing two-times-two tables. True positives were defined as the number referred by GPs with depression and found with depression via MINI interview. A true positive depression could be accompanied by another psychopathology found via MINI. However, depression, as part of a bipolar disorder, was considered a false positive. We used STATA version Stata/SE 15.1 to calculate confidence intervals (command “bootstrap”) in the primary and secondary analyses and to perform summary statistics in the secondary analyses.

## Results

Fifty-one GP clusters from the Collabri trials were randomized according to detection method. Twenty-five were randomized to usual clinical assessment, and twenty-six were randomized to case-finding (Fig. 1). We included a total of 500 participants who were referred by their GP with a depression diagnosis; 256 patients were included in the usual clinical assessment group from a total of 19 GP clusters (mean: 13.5, range: 1:24), and 244 were included in the case-finding group from a total of 19 GP clusters (mean 12.8, range 1:29). Of the 500 participants, 347 were interviewed using the MINI via telephone as this was the mode of administration in the Collabri studies, and 153 were interviewed using the MINI face-to-face as this was the mode of administration in the Collabri Flex studies. Participants in the randomization groups were relatively similar regarding age, gender, and severity of depression, according to MINI (Table 1).

**Table 1:** Baseline characteristics

	Usual clinical assessment, *n *= 256	Case-finding, *n* = 244
Age mean, range	38 (18–81)	40 (18–75)
Male gender, n (%)	96 (38)	88 (36)
GP-diagnosis		
Depression as primary diagnosis, n (%)	231 (90)	218 (89)
Depression as secondary diagnosis, n (%)	25 (10)	26 (11)
MDI available, n (%)	215 (84)	214 (89)
MDI mean sum, score	32	32
Primary diagnosis after MINI interview, n (%)		
Mild depression	30 (12)	24 (10)
Moderate depression	101 (39)	82 (34)
Severe depression	92 (36)	86 (35)
Generalized anxiety, panic disorder or Social phobia	22 (9)	26 (11)
Other diagnosis	4 (1)	11 (4)
No diagnosis	7 (3)	15 (6)
Total	256 (100)	244 (100)

*Abbreviations*: *GP* General practitioner, *MDI* Major Depression Inventory, *MINI* Mini International Neuropsychiatric Interview

The PPV of the depression diagnosis made by GPs in the case-finding group was 0.83 (95% CI 0.78–0.88) and 0.93 (95% CI 0.89–0.96) in the usual clinical assessment group (Table 2). The confidence intervals did not overlap. This indicates a significant difference in favor of the usual clinical assessment group. Thus, findings do not support the hypothesis that using MDI on clinical suspicion improves the precision of the depression diagnosis in primary care, compared to usual clinical assessment, where GPs, if they follow the guidelines, use the MDI or ICD-10 criteria when they have ensured that at least two core symptoms of depression are present. In the usual clinical assessment group, 84% of the GPs presented an MDI sum-score at referral, corresponding to 89% of the GPs in the case-finding group.

**Table 2:** Positive predictive value for depression diagnosis based on case-finding and standard detection

	+ Depression (MINI), n	– Depression (MINI), n	Total, n	PPV (95%CI)
Depression diagnose by usual clinical assessment	237	19	256	0.93 (0.89–0.96)
Depression diagnose by case-finding	203	41	244	0.83 (0.78–0.88)
Total	440	60	500	

*Abbreviations*: *MINI* Mini International Neuropsychiatric Interview, *PPV* Positive Predictive Value

Secondary exploratory analyses show that for the group assessed with a mild depression via MINI interview, the mean MDI sum-score was 26. The group found with moderate depression had a mean MDI sum-score of 31, and the group found with severe depression had a mean MDI sum-score of 36 (Table 3). For the group of participants found with no depression after MINI, the average MDI sum-score was 30.

**Table 3:** MDI sum-score means according to depression severity

Depression degree according to MINI	No. of MDIs	Mean	SD	95% CI for mean
No depression^a^	46	30.2	7.9	27.9–32.5
Mild depression	58	26.4	7.3	24.5–28.2
Moderat depression	164	31.5	6.9	30.4–32.5
Severe depression	159	36.0	5.7	35.1–36.9
Total	427			

Note: Two cases were excluded because of missing data on depression degree according to MINI

*Abbreviations*: *MDI* Major Depression Inventory, *MINI* MINI International Neuropsychiatric Interview

^a^Including cases without a diagnosis or other diagnosis

## Discussion

In this study, case-finding compared to usual clinical assessment did not improve the precision of the depression diagnosis in primary care. If the usual clinical assessment group followed current guidelines, which suggest exploring diagnostic criteria in more detail if two core symptoms of depression were present, the higher PPV could indicate that exploring diagnostic criteria only when suspecting depression is insufficient. This would be a plausible explanation as the prevalence of depression would be higher in a group of patients who the GP suspects have depression and that have at least two core symptoms, compared to patients who the GP only suspects have depression. However, this can only be hypothesized because the approach and usage of MDI in the usual clinical assessment group could vary.

A reason for the unexpected result could also be that GPs in the case-finding group did not fully implement the detection method. GPs in the case-finding group only enclosed an MDI sum-score for 89% of included cases and not for the expected 100%. Unfortunately, we cannot investigate the reason for the lacking MDI sum-scores from the data available. Possibly GPs did not apply the MDI in obvious cases, and greater application of MDI could have given a higher PPV. Research has also shown that GPs could be reluctant to use scales because they feel that the scales do not fit into a fluid conversation, that the results do not always correspond to their clinical impression, and could even be misleading [4]. GPs in the usual clinical assessment group enclosed an MDI sum-score almost to the same extent (84%) as the GPs in the case-finding group. Still, the high usage of MDI in both groups could not explain the higher PPV in the usual clinical assessment group compared to the case-finding group.

The awareness of participating in a study could have influenced the usual behavior of GPs’ in the usual clinical assessment group, but we cannot examine this from the data available. If GPs in the case-finding group applied MDI at a lower threshold of suspicion than normally, they would perhaps detect depression in a sample with a lower prevalence compared to GPs in the usual clinical assessment group. Since PPV depends on prevalence, a reduction would also reduce the PPV. The baseline data did, however, not indicate important differences in characteristics between groups. Perhaps GPs in the usual clinical assessment group had other advantages in their course of detecting depression, e.g., a better prior knowledge of the patient, a different way of dealing with a differential diagnosis, or perhaps they applied MDI with a different timing along the diagnostic process.

The mean MDI sum-scores for each depression severity group according to MINI indicate higher scores than otherwise suggested cut-offs of 21 for mild depression, 26 for moderate depression, and 31 for severe depression [26]. Thus, when using MINI as standard, initial MDI sum-sores presented at referral might have overestimated depression severity in this study sample. However, further studies must be conducted to confirm this finding.

### Strengths and limitations

Strengths of this trial were the relatively large sample of participants, the centralized computer-based cluster-randomization, and the use of blinded assessors regarding the allocation. We consider it as a strength of the study that the MDI was used as an assessment tool. The MDI is a measure already used in general practice and recommended in Danish guidelines [21]. In a study by Nielsen et al., the validity of the MDI, compared against the Munich-Composite International Diagnostic Interview (M-CIDI), was investigated in a set up comparable to usual clinical practice in Danish general practice [27]. Authors found that the MDI was a conservative measure of depression compared to the M-CIDI and a valid tool for diagnosing depression when applied to persons who were suspected to have depression [27]. It was a limitation that we used the MINI as standard reference and not SCID or CIDI. However, we used MINI because it is less time-consuming and a widely used tool for validating symptoms and diagnoses. Performed MINI interviews might not have diagnosed all patients with depression, but if the research assistant’s diagnosis was inconsistent with the GP’s diagnosis, the research assistant consulted a psychiatrist in the Collabri group, who contacted the GP to agree on a result. This procedure would, however, not identify false-positive cases if the MINI would diagnose patients with a depression that did not have depression. The identification of participants relied on the GPs’ judgment, and other recruitment strategies such as identification through records or waiting room screenings could have identified a different sample of participants on which the MDI would have been applied. However, the present strategy of GPs identifying participants is closer to everyday clinical practice. We also cannot rule out the possibility that time between tests may have had an impact, as the symptoms may have fluctuated, or medical treatment could have been initiated.

We planned to estimate the sensitivity, specificity, and NPV of the GP’s depression diagnosis in the case-finding group. However, as GPs only referred few persons believed not to have depression after first suspecting one, we consider these estimates as unreliable. Thus, less information about the case-finding method than first anticipated was gained.

It can impact the external validity that some exclusion criteria from the Collabri trials also applied to this nested study. Moreover, the low number of general practices participating (51 of 713 invited) is also a threat to the external validity, as participating GPs could represent those especially interested in collaborative care or depression detection. Further, if both detection groups perform well, it would be more difficult to detect a difference between groups. It is a limitation that we have no baseline information about GPs on factors such as sex, age, and years of practice, as these could have had an impact on their clinical performance. Additionally, there is a risk that the GP sample size is too small to avoid significant baseline differences between GPs, which could affect the detection practice and thereby, the outcome.

### Comparison with existing literature

The results did not show large problems with false positives in either of the studied groups. In comparison, Mitchell et al. [5] found a PPV of 42.0% (39.6%-44.3%) for the GP depression diagnosis across a sample of 19 studies. In this meta-analysis, depression checklists or questionnaires for GPs were, for example, used to assess the diagnosis. Golden standards were often Structural clinical interview for DSM (SCID), Composite International diagnostic interview (CIDI), and the Diagnostic interview scale (DIS). Christensen et al. found a true positive rate of 60.5% for a group of GPs using case-finding [14]. Still, in our study, 7% of patients in the usual clinical assessment group and 17% of patients in the case-finding group were diagnosed with a depression that was not verified using MINI.

### Implications for research and/or practice

Our findings suggest that usual clinical assessment is more precise than case-finding in this setting. However, more research is needed to support this result. Identification of depression might be improved by integrating the case-finding approach into a stepped care model; however, this should be studied further. Optimally, the MDI should be used for people with at least two of three core symptoms of depression. Thus, it would be relevant to examine the precision of the first three questions in the MDI used as a screening tool before testing with the full MDI.

## Conclusions

In this study, routine clinical assessment outperformed case-finding in the identification of depression in primary care. Further studies should be conducted to confirm this finding.

## Supplementary Information

**Additional file 1: Box 1.** Information about the validation of the MINI interview conducted by telephone.

## Data Availability

Data is not available because of the General Data Protection Regulation. Upon reasonable request data can be retrieved.

## Abbreviations

CIDI: Composite International Diagnostic Interview
GP: General Practitioner
ICD: International Statistical Classification of Disease
MDI: Major Depression Inventory
MINI: MINI International Neuropsychiatric Interview
NPV: Negative Predictive Value
PPV: Positive Predictive Value
RCT: Randomized Controlled Trial
SCID-P: Structured Clinical Interview for DSM-III-Revised, Patients

## References

[1] Olsen LR, Mortensen EL, Bech P (2004). Prevalence of major depression and stress indicators in the Danish general population. Acta Psychiatr Scand.

[2] World Health Organization. Depression and other common mental disorders: global health estimates. Licence: CC BY-NC-SA 3.0 IGO. Geneva; 2017.

[3] Kroenke K (2003). Patients presenting with somatic complaints: Epidemiology, psychiatric co-morbidity and management. Int J Methods Psychiatr Res.

[4] Davidsen AS, Fosgerau CF (2014). What is depression? Psychiatrists’ and GPs’ experiences of diagnosis and the diagnostic process. Int J Qual Stud Health Well-being.

[5] Mitchell AJ, Vaze A, Rao S (2009). Clinical diagnosis of depression in primary care: a meta-analysis. Lancet.

[6] Dowrick C, Buchan I (1995). Twelve month outcome of depression in general practice: does detection or disclosure make a difference?. BMJ.

[7] Thompson C, Kinmonth AL, Stevens L, Peveler RC, Stevens A, Ostler KJ (2000). Effects of a clinical-practice guideline and practice-based education on detection and outcome of depression in primary care: Hampshire Depression Project randomised controlled trial. Lancet.

[8] Hung C-I, Yu N-W, Liu C-Y, Wu K-Y, Yang C-H (2015). The impact of the duration of an untreated episode on improvement of depression and somatic symptoms. Neuropsychiatr Dis Treat.

[9] Siu AL, Bibbins-Domingo K, Grossman DC, Baumann LC, Davidson KW, Ebell M (2016). Screening for depression in adults: US preventive services task force recommendation statement. JAMA.

[10] Thombs BD, Ziegelstein RC, Roseman M, Kloda LA, Ioannidis JPA (2014). There are no randomized controlled trials that support the United States Preventive Services Task Force Guideline on screening for depression in primary care: a systematic review. BMC Med.

[11] Sundhedsstyrelsen. Referenceprogram for unipolar depression hos voksne. 2007. https://www.sst.dk/-/media/Udgivelser/2007/Publ2007/PLAN/SfR/SST_Dep,-d-,rapport,-d-pdf.ashx. Accessed 12 June 2020.

[12] Romera I, Montejo ÁL, Aragonés E, Arbesú JÁ, Iglesias-García C, López S (2013). Systematic depression screening in high-risk patients attending primary care: a pragmatic cluster-randomized trial. BMC Psychiatry.

[13] Baas KD, Wittkampf KA, Van Weert HC, Lucassen P, Huyser J, Van Den Hoogen H (2009). Screening for depression in high-risk groups: Prospective cohort study in general practice. Br J Psychiatry.

[14] Christensen KS, Sokolowski I, Olesen F (2011). Case-finding and risk-group screening for depression in primary care. Scand J Prim Health Care.

[15] Gilbody S, House A, Sheldon T. Screening and case finding instruments for depression. Cochrane Database Syst Rev. 2005. 10.1002/14651858.CD002792.pub2 PMC6769050 16235301

[16] Archer J, Bower P, Gilbody S, Lovell K, Richards D, Gask L, et al. Collaborative care for depression and anxiety problems (Review). Cochrane Database Syst Rev. 2012. 10.1002/14651858.CD006525.pub2 PMC11627142 23076925

[17] Sheehan D, Lecrubier Y, Harnett Sheehan K, Janavs J, Weiller E, Keskiner A (1997). The validity of the Mini International Neuropsychiatric Interview (MINI) according to the SCID-P and its reliability. Eur Psychiatry.

[18] Brinck-Claussen U, Curth NK, Davidsen AS, Mikkelsen JH, Lau ME, Lundsteen M (2017). Collaborative care for depression in general practice: Study protocol for a randomised controlled trial. Trials.

[19] Curth NK, Brinck-Claussen UØ, Davidsen AS, Lau ME, Lundsteen M, Mikkelsen JH (2017). Collaborative care for panic disorder, generalised anxiety disorder and social phobia in general practice: study protocol for three cluster-randomised, superiority trials. Trials.

[20] Curth NK, Brinck-Claussen U, Jørgensen KB, Rosendal S, Hjorthøj C, Nordentoft M (2019). Collaborative care vs consultation liaison for depression and anxiety disorders in general practice: study protocol for two randomized controlled trials (the Danish Collabri Flex trials). Trials.

[21] Dansk Selskab for Almen Medicin. Klinisk vejledning for almen praksis. Unipolar depression. Diagnostik og behandling. 2010. https://www.dsam.dk/files/9/depression_med_links.pdf. Accessed 20 Oct 2020.

[22] Bech P, Rasmussen NA, Olsen LR, Noerholm V, Abildgaard W (2001). The sensitivity and specificity of the Major Depression Inventory, using the Present State Examination as the index of diagnostic validity. J Affect Disord.

[23] Madsen MT, Zahid JA, Hansen CH, Grummedal O, Hansen JR, Isbrand A (2019). The effect of melatonin on depressive symptoms and anxiety in patients after acute coronary syndrome: the MEDACIS randomized clinical trial. J Psychiatr Res.

[24] Okkels N, Jensen LG, Skovshoved LC, Arendt R, Blicher AB, Vieta E (2020). Lighting as an aid for recovery in hospitalized psychiatric patients: a randomized controlled effectiveness trial. Nord J Psychiatry.

[25] Lecrubier Y, Sheehan DV, Weiller E, Amorim P, Bonora I, Sheehan KH (1997). The Mini International Neuropsychiatric Interview (MINI). A short diagnostic structured interview: reliability and validity according to the CIDI. Eur Psychiatry.

[26] Bech P, Timmerby N, Martiny K, Lunde M, Soendergaard S (2015). Psychometric evaluation of the Major Depression Inventory (MDI) as depression severity scale using the LEAD (Longitudinal Expert Assessment of All Data) as index of validity. BMC Psychiatry.

[27] Nielsen MG, Ørnbøl E, Bech P, Vestergaard M, Christensen KS (2017). The criterion validity of the web-based major depression inventory when used on clinical suspicion of depression in primary care. Clin Epidemiol.

