# Supplementary material for: Improving the precision of depression diagnosis in general practice: a cluster-randomized trial
Source: BMC Fam Pract. 2021 May 7;22:88. doi: 10.1186/s12875-021-01432-w (PMC8105936; doi:10.1186/s12875-021-01432-w)
Supplement: Supplementary file 1 — Additional file 1: Box 1. Information about the validation of the MINI interview conducted by telephone. [file 12875_2021_1432_MOESM1_ESM.docx]

Box S1

| To investigate whether we could use the MINI conducted via the telephone, we compared face-to-face interviews with telephone-based interviews. Participants were recruited through the Research Unit for General Practice in Copenhagen and a research project (the Sherpa project) at the Research Unit at Mental Health Center Copenhagen, aiming at people with depression, anxiety, and bipolar disorder.  Participants were 18 years or older, Danish speaking, and gave their oral and written consent. A total of 13 participants were first interviewed by a research assistant using a face-to-face MINI for DSM IV interview. Within five days, another research assistant conducted an equivalent interview over the telephone. Participants were instructed not to reveal the diagnosis from the first interview to the second interviewer. Subsequently, the results of the interviews were compared. We used a Sign-test to test for disagreement between the diagnoses assessed with the two different interview methods. The Sign-test tests the null hypothesis of no agreement (independence) between two results. We found consistency between the two modes of administration in 10 out of 13 cases (p=0.0386), rejecting the null hypothesis of disagreement (independence). |
| --- |
